# Effects of Tempo-Controlled Resistance Training on Corticospinal Tract Plasticity in Healthy Controls: A Systematic Review

**DOI:** 10.3390/healthcare12131325

**Published:** 2024-07-02

**Authors:** Talia Gordon, Michael Jeanfavre, Gretchen Leff

**Affiliations:** Stanford Healthcare, Redwood City, CA 94063, USA; mjeanfavre@stanfordhealthcare.org (M.J.); gleff@stanfordhealthcare.org (G.L.)

**Keywords:** corticospinal excitability, intra-cortical inhibition, primary motor cortex, skill training, strength training, metronome-paced strength

## Abstract

After musculoskeletal injuries, there is often a loss of corticospinal control. Current tendon rehabilitation may not adequately address the corticospinal control of the muscle which may contribute to the recalcitrance of symptom recurrence. This review provides a summary of the current literature regarding the effectiveness of tempo-controlled resistance training (TCRT) in (1) promoting corticospinal plasticity, (2) improving physical performance, and (3) improving strength outcomes in healthy adults. A comprehensive literature search was conducted using electronic databases (PubMed, CINAHL, Embase, and Google Scholar) to identify relevant studies published between 2010 and 2023. Randomized control (RCT) studies that included recreationally trained and untrained healthy adults between 18 and 60 years of age and that compared a TCRT intervention to a control condition were included. Twelve of the 1255 studies identified in the initial search were included in the final analysis. Throughout all included studies, TCRT was shown to elicit greater neural adaptations compared to traditional resistance training methods (i.e., self-paced strength training). These results indicate that TCRT holds promise as an effective method for modulating corticospinal plasticity in healthy adults and may enhance neuromuscular adaptations, including improvements in CSE, decreased SICI, enhanced motor unit synchronization, and voluntary muscle activation.

## 1. Introduction

The corticospinal pathway is a major neural pathway that connects the primary motor cortex to the spinal cord and plays a crucial role in the control and execution of voluntary movements [1]. Deficits in corticospinal excitability (CSE) and inhibition can occur due to various factors, including aging, disuse, or injury, leading to declines in muscle strength, power, and functional abilities [1,2].

Corticospinal excitability refers to the responsiveness or excitability of the primary motor cortex, which is responsible for generating voluntary muscle movements and is vital for motor control, motor learning, and the acquisition of new skills [3]. Short-interval intracortical inhibition (SICI), on the other hand, is a mechanism by which the brain maintains balance between excitatory and inhibitory signals within the motor cortex. It involves the inhibition of certain neural pathways, preventing excessive neuronal firing and ensuring precise and controlled muscle movements [4]. The clinical effects of intra cortical inhibition in runners with Achilles tendinopathy was observed, and demonstrated that participants with tendinopathy exhibited greater triceps surae intra-cortical inhibition and subsequently decreased endurance based on a single leg heel rise test compared to healthy controls [5]. This demonstrates that weakness and inhibition of the muscle may be linked to the neuronal changes observed in those with chronic musculoskeletal conditions. Thus, ensuring appropriate corticospinal responses after injury as well as including increased CSE and decreased SICI is integral, as these are fundamental components of motor learning, motor control, adaptation, and rehabilitation [2]. 

Over the past several decades, transcranial magnetic stimulation (TMS) has gained popularity in the evaluation of motor cortical reorganization and neuroplasticity [6]. TMS is a non-invasive medical procedure that involves the use of electromagnetic fields to stimulate specific areas of the brain. It is primarily used for therapeutic and diagnostic purposes in the field of neuropsychiatry and neurology [7]. TMS has been demonstrated to be a reliable and valid means to objectively quantify CSE and SICI in active adults [8]. The studies included in this review utilized TMS as an objective measure to quantify changes in CSE and SICI when skilled training is externally paced using a metronome.

It has been described that the motor cortex and spinal cord hold the miraculous ability to alter the structure and function in response to differential motor training [9]. Pertinent to the current study, strength training has recently been considered a form of motor training or motor learning [10,11]; however, the type and magnitude of adaptation within the corticospinal tract is largely dependent on the type of motor training performed [9]. Specifically, skill training induces synaptogenesis, synaptic potentiation, and the reorganization of movement representations within the motor cortex [12]. The acquisition of skilled movement induces a reorganization of neural circuitry within the motor cortex that supports the production and refinement of skilled movement sequences. This evidence supports the idea that the corticospinal system is not only plastic but that the nature and locus of this plasticity are dictated by the specifics of the motor experience [12]. The literature also suggests that increases in strength may be mediated by an increased capacity for the activation and/or recruitment of spinal motoneurons [9]. 

Several factors and movement-based strategies can facilitate such neuroplastic changes. The incorporation of tempo-controlled resistance training (TCRT) has gained attention as a potential strategy to optimize corticospinal plasticity by increasing excitability while reducing intracortical inhibition to enhance neuromuscular adaptations within the primary motor cortex [12,13,14]. TCRT, unlike traditional self-paced strength training, is a method of strength training that involves intentional manipulation of the speed or tempo at which exercises are performed using an external metronome. By manipulating the tempo, this allows for more precise control over the speed of each repetition and can provide additional challenges or stimuli to the primary motor cortex, leading to improvement in overall performance and motor control. The identified TCRT influences on the central nervous system (CNS) have significant implications for motor learning, as it suggests that synchronizing motor training tasks with an audible or visual cue serves as an external stimulus that appears necessary for inducing task-specific adaptation within the corticospinal tract. Furthermore, it has been shown that, at the cortical level, adaptive changes in the primary motor cortex (M1) might also contribute to the early phase of strength development [2]. It has been described that the neural adaptations to strength training may be due to changes in corticospinal excitability and inhibition, and that such changes contribute to the gain in strength following strength training [2]. 

It was found that metronome-paced strength training can reduce SICI and increase CSE compared to self-paced strength training in healthy individuals, as traditional self-paced strength training does not sufficiently alter muscle coordination, motor unit recruitment, and neuromuscular adaptations [12,14]. Additionally, TCRT has been demonstrated to be effective in what is known as the crossover phenomenon, which states that training in one limb can lead to changes in strength and/or corticospinal responses in the contralateral limb [14]. This is especially significant for clinicians as it provides an opportunity to utilize and train the contralateral limb during rehabilitation when the ipsilateral limb is too painful or inhibited in movement secondary to musculoskeletal or neurological injury. 

Thus far, investigations into corticospinal plasticity have solely focused on healthy individuals [12,15,16,17,18,19,20,21,22,23,24,25]. Further examination is required to ascertain the impact of TCRT and the modulation of the corticospinal tract in individuals with musculoskeletal disorders. Despite this, current research has shown that, in healthy subjects, the use of an external metronome to pace resistance training compared to self-paced strength training has consistently demonstrated increases in CSE and reductions in SICI, indicating corticospinal plasticity [12,15,16,17,18,19,20,21,22,23,24,25]. 

The purpose of the current systematic review was to identify the effects of TCRT on corticospinal plasticity in healthy adults. A secondary outcome of this review was to determine the short-term effects of strength after TCRT. The findings will contribute to the growing body of evidence supporting the integration of TCRT as a potentially effective strategy for modulating neural adaptations within the corticospinal tract and primary motor cortex. By enhancing our understanding of these effects, this research may pave the way for optimized exercise interventions that promote neuroplasticity and ultimately improve motor function in both healthy individuals and clinical populations alike. 

## 2. Materials and Methods

The current systematic review was conducted according to The Preferred Reporting Items for Systematic Review and Meta-Analysis (PRISMA) guidelines [26]. The current review sought to identify the effects of TCRT on corticospinal tract plasticity in healthy controls.

### 2.1. Study Identification and Search Strategy

Relevant articles were identified by searching PubMed, CINAHL, Embase, and Google Scholar to identify relevant studies published between January 2010 and May 2023. The search strategy was generated using Zotero and was audited by a medical school librarian to ensure the appropriate use of Boolean modifiers, accurate translation of the search strategy across databases, and the appropriateness of the search based on the study’s stated purpose. The keywords used were variations and derivatives of the following: “[corticospinal plasticity]”, “[tempo-controlled strength training]”, and “[metronome paced strength training]”. Additionally, to ensure a comprehensive identification process, hand-selected articles that were identified through the study selection process or by scouring the references of the included articles were also included.

### 2.2. Eligibility Criteria

The research question was initially framed using the recommended PICO format [26,27]. The PICO question variables, study elements, and the respective inclusion and exclusion criteria are outlined in Table 1.

### 2.3. Study Selection

**Figure 1:**
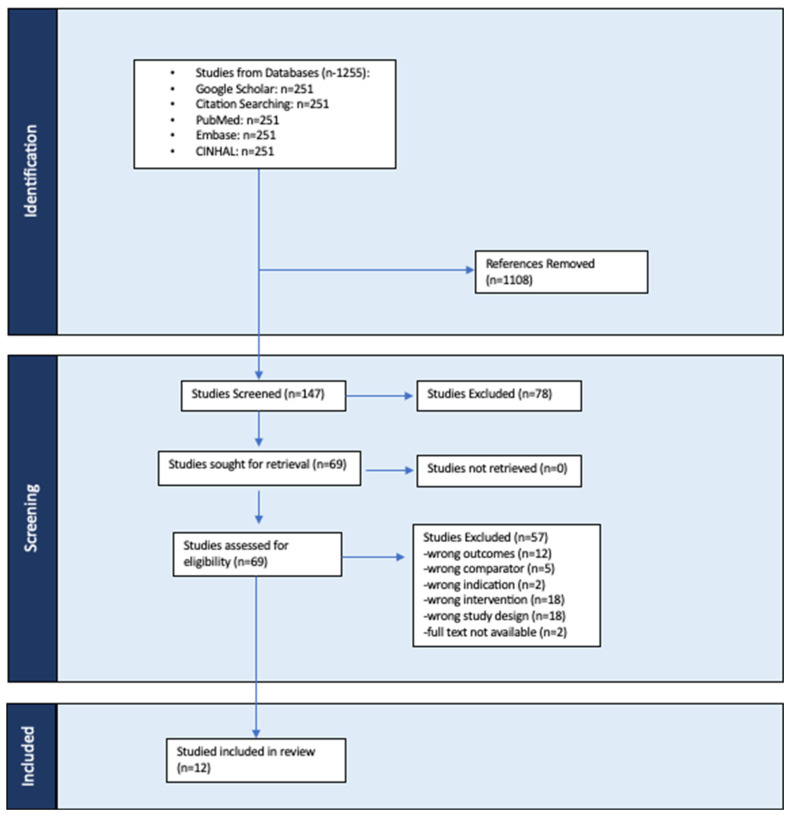
The PRISMA Flow Diagram.

The initial search results of the different databases were combined, duplicates deleted, and filtered independently according to the specified inclusion and exclusion criteria using a citation manager, Zotero, and a systematic review software management system, COVIDence (Veritas Health Innovation, Melbourne, Australia). Figure 1 outlines the study selection process in a PRISMA flow diagram. Upon the initial search, 8 articles were found using CINAHL, 18 articles were found using Embase, 71 articles were found through Google Scholar, and 85 articles were found through PubMed. 

### 2.4. Data Extraction

Data elements of identified full-text articles were prospectively determined based on the PICO question and the purpose of the current study. For each study, data extraction was based on the following characteristics: (1) last name of author and year of publication, (2) study design, (3) sample size, (4) intervention, (5) subject demographics, (6) outcomes, (7) exclusion criteria, and (8) methods. Please refer to Figure 1 for a summary of study characteristics and Table 2 for study qualifiers. 

The level of evidence for all included studies was assessed according to criteria adapted from the Centre for Evidence-Based Medicine, Oxford, United Kingdom [28]. The OCEBM tool utilizes study design, randomization, blinding, and the quantity of bias to grade studies on a scale from I–V, with I being the highest level of evidence. A summary of the OCEBM criteria for each level of evidence is provided in Appendix B.

## 3. Results

Upon the initial search, 1255 articles were identified. After the removal of duplicates, 147 articles remained. Of the 147 articles, 133 were excluded for various reasons. This resulted in the remaining 14 articles which were read in full. Amongst these, 12 were deemed appropriate for final analysis. All 12 included studies were published between 2011 and 2020 involving a total of 332 healthy, untrained adult participants (173 males, (52%) and 161 females (48%) with the mean cohort age, ranging from 18–49 years old. Study characteristics included authors and the year of publication, the methodology by which the test was performed, the interventions included, the measurement property, and the quality of the measurement property, as demonstrated through the PEDro scale. 

Change in CSE was assessed in all 12 studies, while 11 studies assessed the change in SICI after training, and seven studies assessed the changes in strength after training. Throughout all 12 studies, an increase in CSE and a decrease in SICI were found, where CSE and SICI were deemed unchanged within the control or self-paced training groups. Strength increased throughout both metronome-paced and self-paced strength training groups; however, a statistically significant (*p* < 0.05) difference between groups, with a more significant increase in strength amongst the intervention group, was found. A detailed breakdown of interventions and treatment parameters can be found in Appendix A. Included is also a weighted average of change regarding SICI and CSE. This method ensures that values with higher weights contribute more to the final average, reflecting their relative importance to the dataset. This average was calculated by multiplying each value in a dataset by its corresponding weight, summing these products, and then dividing by the sum of the weights. 

### 3.1. Intervention Training Parameters

Throughout the 12 articles included, eleven studies used four sets of 6–8 repetitions with a metronome-paced tempo ranging from 2–3 s for the concentric phase and 2–4 s for the eccentric phase [12,16,17,18,19,20,23,24,25]. The remaining study performed 120 repetitions of an isotonic movement at a pace of 800 Hz with a 50 ms pulse duration [15]. The resistance utilized across studies ranged from 20–80% of 1-RM. Four studies performed the training for a duration of 3×/week for 3 weeks [16,17,20,21], four studies at a duration of 3×/week for 4 weeks [12,19,24,25], three studies only performed the training for a single session [18,22,23], and one study performed the training for 3 sessions, 24 h apart [15]. Of the studies included, 83% (*n* = 10) assessed upper extremity resistance training, while only 17% (*n* = 2) assessed lower extremity resistance training.

#### 3.1.1. Corticospinal Excitability 

Through the use of TMS, CSE increased throughout the metronome-paced strength training groups from a range of 15% to 116% with a weighted average of 60.2% increase. All 12 studies demonstrated unchanged CSE of the self-paced control group. 

#### 3.1.2. Short-Interval Cortical Inhibition 

Through the use of TMS, SICI was assessed. Across the 12 studies included, reductions in SICI ranged from 15.3% to 61% with a weighted average of 40.5% decrease. All 12 studies included demonstrated unchanged SICI of the untrained control group. 

#### 3.1.3. Strength 

Seven (58%) of the studies included strength as an outcome. Strength was measured via 1-RM before and after metronome-paced strength training compared to the control. The increases in strength ranged from 18% to 41% 1-RM following metronome-paced strength training with a weighted average of 25% 1-RM. The self-paced strength training groups exhibited an increase in strength as well, which ranged from an increase of 15% to 35% 1-RM with a weighted average of 19% 1-RM. Although average increases in strength between groups were similar, six out of the seven studies demonstrated a clinically significant (*p* < 0.05) improvement in strength of the metronome-paced strength training group compared to the self-paced strength training group [12,16,17,20,21]. 

#### 3.1.4. Risk of Bias Assessment 

Consistent with the Cochrane Handbook, the risk of bias (RoB) and quality appraisal of the included RCTs were assessed [29]. The RoB assessment of the included studies was performed using the PEDro scale which is summarized in Table 2. All the RCTs were deemed to be “good” with a low risk of bias. 

#### 3.1.5. Level of Evidence 

According to the OCEMB 2011 Levels of Evidence, the majority of studies were determined to be of Level 2 Quality as all 12 studies were randomized control trials. 

## 4. Discussion

The purpose of this systematic review was to determine the effects of TCRT on corticospinal adaptations in healthy adults. This tested the hypothesis that the intracortical excitatory and intracortical inhibitory circuitry of the M1 would adapt depending on the strength training task being performed. The findings of this systematic review confirm this hypothesis, whereby performing TCRT induced task-specific adaptations in CSE and SICI compared to self-paced strength training. Across the 12 articles that were included, TCRT compared to the self-paced strength training group consistently demonstrated increases in CSE and decreases in SICI, thus exhibiting functional changes in the strength of corticospinal projection and potentially indicating the subsequent reorganization of cortical mapping within the primary motor cortex. 

These findings were based on studies that were deemed to be of level II quality evidence and low risk of bias (i.e., “good”), according to the Oxford Center for Evidence-Based Medicine and PEDro scale, respectively. Of the 12 studies included, the mean PEDro score was 7.16/10 with a range of 6–8/10. Interestingly, 75% of the studies failed to incorporate the blinding of assessors and subjects. However, with the intervention included within this review, it would be challenging to blind subjects to either a control or intervention group. Thus, blinding assessors may have provided higher-quality evidence. 

The summative results of this review demonstrated a weighted mean increase in CSE of 60.2% and a decrease in SICI of 40.5% across the 12 studies, with 100% of the included studies demonstrating no change in CSE or SICI for the self-paced or control groups. This has supported the idea that the corticospinal system is not only plastic but that the nature and locus of this plasticity are dictated by the specifics of the motor experience. It demonstrates that with specific skilled training, re-organization within the M1 takes place. Despite consistent increases in CSE compared to the control, the data ranged from an increase of 15% to 116% throughout the 12 included studies. The variation may be attributed to training employed, including a heavy training load versus a light load as well as progressive overload throughout the training sessions. The complexity of movement may have also attributed to the variation in CSE, as heavy loads and complex movement patterns are known to induce muscle stiffness by facilitating sensorimotor input into the M1 [30]. This hypothesis was tested further by incorporating an isometric training group who performed the strength training intervention while listening, but not pacing to a metronome [24]. Unlike the true metronome paced strength training (MPST) group, this group demonstrated no changes in CSE or SICI [24], further demonstrating that corticospinal responses are task-dependent and require a novel or challenging task to be placed upon the corticomotor system. The neural mechanisms responsible for this task-dependent change following MPST are likely due to reduced inhibition and increased recurrent excitation within the intracortical circuits responsible for controlling the trained muscle. When performing slower and paced repetitions, it is likely that an increase in precision was required to maintain the specific timing of the movement, thus strengthening existing neural connections and potentially the formation of new connections via the removal of local inhibition, leading to greater levels of use-dependent plasticity [15].

A subsequent finding was the short-term increase in strength that was demonstrated after metronome-paced strength training. In this review, seven out of the 12 studies included strength via 1-RM as an outcome. Of the seven studies, six (86%) exhibited statistically significant improvements (*p* < 0.05) in 1-RM strength when comparing metronome-paced strength training to self-paced strength training. Additionally, the MPST group exhibited a weighted increase of 25% 1-RM, while the self-paced group exhibited a weighted increase of only 19% 1-RM across studies. This finding is consistent with past research and demonstrates that changes in strength may be directly related to the neuronal changes that were observed [12]. As strength training can be a form of motor learning when a novel and complex task is performed, it is then perceived that plastic changes may occur within the CNS at both a cortical and spinal level following motor training. This provides evidence that both the primary motor cortex and the corticospinal tract work as a dynamic and integrated neural network to execute the required muscle contractions during specific strength training interventions [20]. 

Additionally, there was likely a degree of specificity between the self-paced strength training task and the 1-RM strength test, as these were performed and tested without the use of a metronome. This explains, then, why strength improved as much as it did within the self-paced strength training groups. 

It is important to also discuss the acute corticospinal responses to strength training immediately after a single session versus the longer-term corticospinal adaptations following multiple weeks of training that were observed throughout the included studies. One study directly examined the temporal dynamics of strength training in fostering use-dependent cortical plasticity and its implications for augmenting muscular strength [11]. The main findings indicate that the M1 undergoes substantial use-dependent plasticity from the first strength training session onwards alongside a reduced co-contraction of antagonists to drive improvements in muscular strength. These adaptations are rapid, and beyond the immediate cellular response to the initial strength training session, occur primarily between strength training sessions, and culminate in longer-term functional changes (i.e., neurogenesis). Understanding how the brain adapts to strength training can help clinicians design more effective rehabilitation programs. By tailoring training sessions to maximize these rapid neural adaptations, therapists can potentially enhance recovery outcomes for patients recovering from musculoskeletal injuries or neurological conditions.

Additionally, although not a primary finding of this review, what is known as the crossover phenomenon was observed when metronome-paced strength training was performed in the contralateral limb. Cross-education is defined as the improvement in motor performance of an untrained limb following a unilateral motor training program and can include the transfer of muscular strength and/or the transfer of motor skills [19]. This is known to occur when there is concurrent activation of both cerebral cortices which can be seen during both skill and strength training [12]. This was assessed in five out of the 12 studies included in this systematic review and included the assessment of the ipsilateral corticospinal response of the contralateral limb following different types of unilateral motor training. In one study, corticospinal responses of the ipsilateral limb were compared following MPST, visuomotor skill training (VST), and self-paced strength training [19]. Only the MPST and VST groups observed an increase in CSE and a decrease in SICI, with the MPST group exhibiting the greatest change. As stated previously, this is thought to be mediated by the functional changes (plasticity) and neural adaptation occurring within the primary motor cortex (M1) which is enhanced through skill training, inducing synaptogenesis, synaptic potentiation, and the reorganization of movement representation maps in the contralateral and ipsilateral M1 and is highly dependent on the type of motor training being performed [19]. These findings provide insight into ways to maximize clinical potential by utilizing cross-education to increase the magnitude of the contralateral increase in motor function especially in the presence of asymmetric conditions. 

Though the results of the current review provide a novel training strategy with which health, fitness, strength, and conditioning practitioners might induce more robust long-term changes in motor learning in their clients and athletes, evidence is lacking on the effect of TCRT on corticospinal plasticity in a clinical or injured population. The data extrapolated from this review provides convincing evidence for the potential to enhance traditional rehabilitation with the addition of TCRT in individuals with musculoskeletal injuries.

One such example of a clinical population who demonstrates altered CSE and SICI are persons diagnosed with tendinopathies [5,13,14,31]. Pathologically, tendons exhibit changes in their anatomical structure, including enlarged and increased numbers of tenocyte cells, disrupted collagen organization, higher levels of proteoglycan and water, and the development of new blood vessels [32]. Alongside the structural, functional, and biomechanical alterations observed in tendinopathy, recent evidence also has revealed modifications in the motor cortex of individuals affected by this condition. Specifically, measurable variations in CSE and SICI have been identified. [13,14]. The mechanisms that contribute to Achilles tendinopathy have been investigated, as these remain poorly understood [31]. The disparity between pain experience and peripheral pathology demonstrated in patients with Achilles tendinopathy suggests that changes in central nervous system function may be involved. As past research has demonstrated, tendon load may be influenced by both volume and quality of loading [33]. There is growing evidence to suggest that movement quality may be impacted by impaired peripheral factors such as strength and tendon stiffness in those with tendon pain [34]. However, less attention has been paid to central mechanisms that contribute to movement quality, particularly centrally stored maps of the body that allow for the accurate planning, coordination, and execution of movement [31]. 

**Figure 2:**
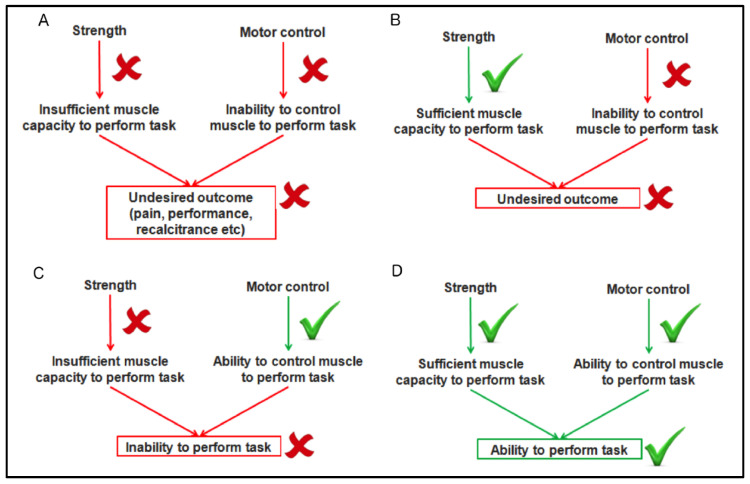
Summary of different approaches of tendon rehabilitation, and effects on strength and motor control. Images (**A**–**D**) denoting outcomes based on inclusion of strength and motor control in tendon rehabilitation. Note: Reproduced from [13] with permission from BMJ Publishing Group Ltd.

Current rehabilitation protocols may not adequately address the corticospinal control of the muscle; this may result in altered control in muscle recruitment and consequent tendon load, which may contribute to recalcitrance or symptom recurrence [13,14] (Figure 2). 

Potential alterations in central motor representation in individuals with chronic RTC tendinopathy have also been investigated [35]. Findings revealed that individuals with RTC tendinopathy exhibited significant alterations in central motor representation in the infraspinatus secondarily to a decrease in excitability and an increase in inhibition [35]. Understanding the involvement of the CNS in musculoskeletal disorders should be considered as a key aspect to improve the management of patients with such disorders [35]. Taking the results of the current review, the identified mechanisms of the action of TCRT, as well as emerging, yet limited, evidence of the positive effects that metronome-paced strength training MPST can have on pain and function in those with chronic musculoskeletal disorders (i.e., chronic tendinopathy), there is exciting potential for rehabilitation professionals to not only restore the mechanical properties of the peripheral contractile tissues but to also restore the CNS alterations that accompany musculoskeletal injuries. 

Several studies have reviewed the corticospinal adaptations to motor skills and resistance training [2,9,36,37,38,39], and found a positive correlation with skilled tasks and motor learning; however, to the best of the author’s awareness, this is the first systematic review to date that specifically examines the current effectiveness of TCRT on CNS neuroplasticity (CSE and SICI), motor learning, and muscle performance. Other studies have recognized the clinical importance of the further examination of novel techniques including TCRT to evoke neural adaptations [13,39]. All studies included in this review support the notion that externally paced strength training, which is akin to a skilled movement task, has been shown to not only reduce tendon pain but modulate excitatory and inhibitory control of the muscle and, therefore, potentially tendon load. An improved understanding of the methods that maximize the opportunity for neuroplasticity, such as the ones described in this review, may offer an alternate framework for how rehabilitation and strength and conditioning professionals prescribe exercise-based interventions in tendinopathy for pain modulation and potentially the restoration of the corticospinal control of the muscle–tendon complex.

Although this systematic review has identified that performing metronome-paced resistance training induced task-specific adaptations in CSE and SICI compared to self-paced strength training, there are limitations that must be considered when interpreting these findings. Firstly, there was little discrepancy amongst the metronome-paced strength training protocols utilized within the included studies, which restricted the exploration of different parameters and their potential impact on corticospinal plasticity. Future research should consider utilizing a wider range of parameters including load, duration, metronome pacing, and intensity to identify the optimal TCRT prescription and dosage. Secondly, there was the lack of a specific self-paced strength training comparison group, versus simply a control group in a multitude of studies in which the control group completed their normal strengthening exercise without following any specific protocol. Including such a group would have provided clearer and more consistent evidence regarding the advantages of TCRT compared to traditional strength training methods. More research should be carried out on this topic to identify the effects of TCRT on those with musculoskeletal injuries to broaden the applicability of the findings to a clinical population. 

## 5. Conclusions

The current findings identify that there is a current level II quality of evidence that there is a strong acceptance of the hypothesis that, in the healthy adult, performing TCRT can induce task-specific adaptations in CSE and SICI compared to self-paced strength, thus eliciting adaptations within the primary motor cortex. This has been compared mostly to self-paced or non-skilled strength training. The increase in CSE and decrease in SICI is thought to be mediated by use-dependent plasticity within the primary motor cortex after a skilled training task is performed. This evidence has shown that movements that are synchronized to a metronome or that are more skillful lead to specific activation within the sensorimotor cortex, premotor cortex, and supplementary motor area. It was found that strength was also improved following MPST which suggests that strength changes may be directly related to the neuronal adaptations observed. The findings in this review also suggest that future research should explore the application and efficacy of TCRT interventions in clinical populations. The application of such interventions has the opportunity to not only induce the musculotendinous structural and performance changes seen following traditional (i.e., self-paced) exercise-based interventions but to induce more permanent adaptations within the CNS via neuroplasticity which, in turn, enhance motor performance. Future research should seek to identify the optimal TCRT prescription and dosage by comparing a spectrum of frequency, tempo, intensity, variability, duration, and volume of training variables. Considering the newfound appreciation for the role of the reticulospinal tract in strength execution, coordination, and motor neuron facilitation, the authors encourage future researchers to also investigate the influence of timed strength training on the reticulospinal tract. 

## Figures and Tables

**Table 1:** PICO Question and Study Design Inclusion and Exclusion Criteria.

Question Component	Question Content	Inclusion Criteria	Exclusion Criteria
Population	Healthy Individuals, age 18–60	Population must include recreationally trained and untrained healthy, young humans of either gender between the ages of 18–60 years old	Individuals must be free of any known neurological disorders
Intervention	Tempo-Controlled Resistance Training	Studies must have compared an intervention to a control condition	Any diseased populations
Comparison	Other conservative treatment, self-paced strength training	Studies must include tempo-controlled resistance training as an intervention	No previous surgery to the body segment involved
Outcome	Corticospinal Plasticity	There must be a quantifiable measurement of corticospinal excitability and inhibition	Include all experimental study design but exclude expert opinion (Grey literature)
Study Design	RCT’s	English Language	Articles that do not have full text available

Note: RCT’s, randomized control trial; y/o, years old.

**Table 2:** Summary of Risk of Bias Assessment for Randomized Control Trials.

PEDro Scale	Mason and Frazer et al., 2020 [11]	Leung et al., 2017 [12]	Ackerly et al., 2011 [15]	Coombs et al., 2016 [16]	Goodwill et al., 2012 [17]	Leung et al., 2015 [18]	Leung et al., 2018 [19]	Mason et al., 2017 [20]	Mason and Frazer et al., 2019 [22]	Mason and Howatson et al., 2019 [23]	Siddique et al., 2020 [24]	Weier et al., 2012 [25]
1. Eligibility criteria	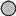	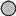	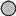	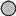	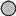	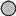	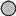	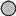	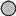	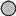	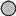	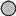
2. Random allocation	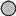	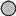	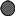	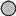	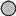	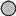	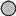	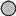	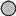	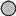	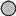	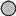
3. Concealed allocation	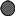	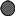	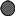	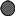	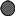	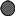	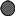	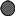	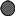	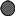	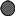	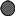
4. Baseline comparability	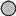	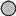	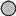	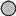	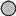	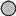	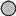	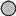	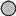	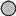	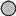	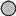
5. Blind subjects	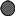	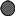	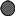	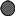	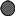	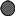	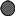	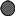	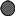	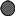	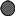	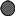
6. Blind therapists	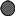	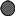	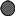	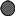	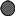	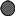	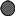	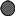	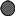	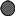	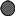	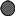
7. Blind assessors	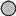	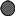	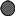	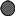	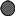	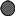	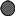	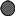	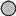	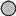	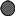	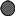
8. Adequate follow-up	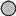	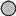	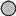	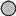	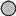	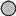	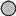	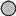	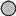	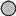	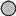	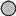
9. Intention-to-treat analysis	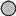	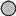	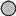	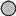	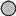	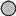	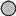	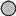	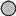	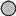	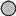	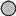
10. Between group comparisons	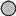	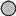	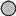	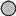	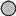	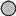	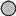	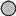	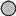	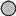	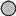	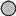
11. Point estimates and variability	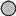	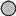	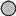	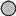	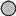	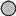	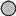	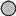	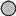	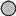	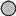	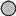
Total	8/10	7/10	6/10	7/10	7/10	7/10	7/10	7/10	8/10	8/10	7/10	7/10
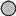 = criteria met 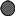 = criteria not met												

Note. Scoring: 0–3 = poor, 4–5 = fair, 6–8 = good, 9–10 = excellent.

**Figure A1:**
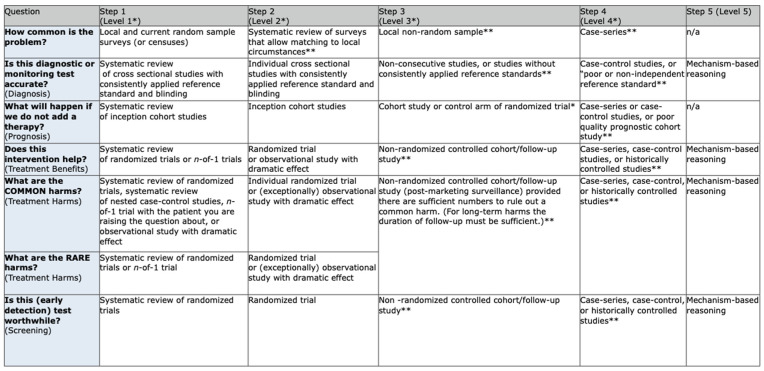
Level of evidence from Oxford Centre for Evidence-Based Medicine 2011 Levels of Evidence [28]. (* Level may be graded down on the basis of study quality, imprecision, indirectness (study PICO does not match questions PICO), because of inconsistency between studies, or because the absolute effect size is very small; Level may be graded up if there is a large or very large effect size. ** As always, a systematic review is generally better than an individual study. n/a: Not Applicable).

## Data Availability

Not Applicable.

## Appendix A

**Table A1:** Data Elements of Included Studies.

Study	Design	Evidence Level	Intervention Groups	Parameters	Sample Size	Age Mean ± SD	Key Measures	Results	PEDro Score
Mason and Frazer et al., 2020 [11]	RCT	II	Intervention: right elbow flexion and extension at 4 sets × 6–8 reps at 80% of 1-RM of unilateral dynamic elbow flexion. Tempo: 3″ concentric—4″ eccentric. 3 min rest between sets Control group: No training	Training group: 3 weeks strength training of right biceps brachii	n = 20, F = 10, M = 10	18–35 years old	CSE and SICI via TMS, strength via 1-RM	Intervention: strength ↑ 29%, CSE ↑ 49%, SICI ↓ 17.5% Control: CSE ↓ 2%, SICI ↓ 1%	8
Leung et al., 2017 [12]	RCT	II	Skill training: 4 × 56 s of visuomotor tracking through range of 30° to 140° moving within a 0.2–1.3 Hz range. 3 min recovery between sets. MPST: 4 sets × 6–8 reps at 80% of 1-RM. Tempo: 3 s concentric—4 s eccentric. 3 min recovery between sets. SPST: 4 sets × 6–8 reps at 70–80% of 1-RM. Tempo: self paced. 3 min recovery between sets. control group: no training	All training groups: bicep curls of dominant arm 3×/week for 4 weeks	N = 44; F = 20, M = 24	26.1 ± 6.8 years	CSE, SICI, strength, and tracking error at 2 and 4 weeks via TMS and 1-RM/MVC	Skill training: strength ↔, CSE ↑ 29%; SICI ↓ 41% and 33% following 2 and 4 weeks, tracking error ↓ 58% at 4 weeks MPST: strength ↑ 7 and 18% @ 2 and 4 weeks; CSE ↑ 40%; SICI ↓ 41 and 61% following 2 and 4 weeks, tracking error ↓ 24% at 4 weeks SPST: ↑ strength by 12 and 26% @ 2 and 4 weeks, SCE and SICI ↔, tracking error ↓ 13% control: ↔ in strength, CSE, or SICI, tracking error ↓ 14%	7
Ackerly et al., 2011 [15]	RCT	II	Metronome paced: 120 wrist extensions, self paced with metronome AND increased demand pace (1.5× faster than SP) Self paced: 120 wrist extensions, self paced no metronome, 30 s rest period	All training groups: measure of wrist extension, 3 sessions, 24 h apart	n = 12; F = 6, M = 6	mean age 26 years [range 20–49]	corticomotor excitability via TMS	significant increase in ECR MEP area/corticomotor excitability (*p* = 0.023) only after MP training	6
Coombs et al., 2016 [16]	RCT	II	RHT and LHT groups: 3×/week for 3 weeks, 4 sets × 6–8 reps at 70% of 1-RM with 3 min recovery. Tempo: 3″ concentric—4″ eccentric Control group: Control period without training	Training: 3×/week for 3 weeks of right handed or left handed strength training of the wrist extensors	n = 23, F = 12, M = 11; all right hand dominant	18–36 years	CSE and SICI via TMS and strength via 1-RM, and silent period	Trained limb: 22% ↑ strength, CSE and SICI ↓ at higher TMS intensities Untrained limb: 15% ↑ strength, CSE and SICI ↔, SP ↓ 47 ms	7
Goodwill et al., 2012 [17]	RCT	II	Strength training group: 4 sets × 6–8 reps at 75–80% of 1-RM of single leg squats. Tempo: 3″ concentric—4″ eccentric Control group: Control period without training	Training group: 3×/week for 3 weeks of right SL squats	n = 14, F = 7, M = 7	18–35 years	CSE and SICI of the untrained and trained limb via TMS, strength via 1-RM	Intervention: RLE strength ↑ 41%, RLE CSE ↑ 55%, RLE SICI ↓ 24.5%, LLE strength ↑ 35%, LLE CSE ↑ 62%, SICI LLE ↓ 21% Control: CSE and SICI: ↔	7
Leung et al., 2015 [18]	RCT	II	MPST: Dominant elbow flexion: 4 sets × 6–8 reps at 70–80% of 1-RM. Tempo: 3 s concentric—4 s eccentric SPST: Dominant elbow flexion: 4 sets × 6–8 reps at 70–80% of 1-RM. Tempo: Preferred tempo Skill Training: 4 × 56 s of visuomotor tracking through 120° range moving within a 0.2–1.3 Hz range. 3 min recovery between sets. Control group: Without training	All training groups: bicep curls of dominant arm for a single training session	n = 44; F = 20, M = 24	26.1 ± 6.8 years	CSE and SICI via TMS	MPST: trained arm: CSE: ↑ 43.3 ± 4.9% SICI: ↓ 34.2% ± 2.5; untrained arm: CSE ↔, SICI ↓ 20.3% SPST: trained and untrained arm: CSE and SICI: ↔ Skill training: trained arm: CSE ↑ 41.8% ± 5.7, SICI↓ 33.1% ± 2.9; untrained arm: CSE ↔, SICI ↓ 29.2% Control: trained and untrained arm: CSE and SICI: ↔	7
Leung et al., 2018 [19]	RCT	II	Metronome paced group: 4 sets × 6–8 reps at 80% of 1-RM of unilateral dynamic elbow flexion. Tempo: 3″ concentric—4″ eccentric with metronome, 3 min recovery between sets Self-paced group: 4 sets × 6–8 reps at 80% of 1-RM of unilateral dynamic elbow flexion. Preferred tempo, no metronome. 3 min recovery between sets visuomotor skill training: 4 × 56 s of visuomotor tracking through range of motion from 30° to 140° at a rate of 0.2–1.3 Hz Control group: Control period without training	All training groups: 3×/week for 4 weeks of right elbow flexion	n = 43; F = 22, M = 23	26.4 ± 6.9 years	CSE and SICI of the untrained limb via TMS, strength via 1-RM	MPST (untrained): strength ↑ 8 and 16% at 2 and 4 weeks, CSE ↑ 89 and 105% at 2 and 4 weeks, SICI ↓ 45 and 48% at 2 and 4 weeks MPST (trained): strength ↑ 28 and 26% at 2 and 4 weeks SPST (untrained): strength ↑ 6 and 13% at 2 and 4 weeks, CSE and SICI: ↔ SPST (trained): strength ↑ 28 and 26% at 2 and 4 weeks, CSE ↔ Skill training: strength ↑ 47 and 58% at 2 and 4 weeks, CSE ↑ 71 and 81% at 2 and 4 weeks in the untrained limb, SICI ↓ 47 and 38% at 2 and 4 weeks in the untrained limb Control: CSE and SICI: ↔	7
Mason et al., 2017 [20]	RCT	II	Trained: flexion-extension of the right elbow: 4 sets × 6–8 reps at 80% of 1-RM. Tempo: 3″ concentric—4″ eccentric. 3 min recovery between sets Control: no training	Training group: 3 weeks strength training of right biceps brachii	n = 20, F = 10, M = 10	18–35 years	CSE and SICI of the untrained limb via TMS, and strength via 1-RM	Trained biceps brachii: strength ↑ 29% Untrained biceps brachii: strength ↑ 23%, CSE ↑ 25%, SICI ↓ 15.3% Control: 1% ↑ CSE, SICI 1% ↓	7
Mason and Frazer et al., 2019 [22]	RCT	II	Skill training: 4 × 56 s of visuomotor tracking 30° to 140° moving within a 0.2–1.3 Hz range heavy-load: 4 × 6–8 reps at 80% 1 RM with 2.5 min of rest between sets. Tempo: 2 s concentric; 4 s eccentric light-load: 4 × 20 reps at 20% 1 RM with 30 s rest between sets. Tempo: 2 s concentric; 4 s eccentric Control: No training	Training groups: single session of either low load, high load, or skilled unilateral biceps curls	n = 40, F = 20, M = 20	25.9 ± 6.4 years	CSE and SICI via TMS	Skill training: CSE ↑ 38%, SICI ↓ 47% heavy load: CSE ↑ 37%, SICI ↓ 40% Light load: CSE ↑ 46%, SICI ↓ 4% Control: CSE ↑ 3.72%, SICI ↓ 1.36%	8
Mason and Howatson et al., 2019 [23]	randomized, counterbalanced, cross-over design	II	Heavy load strength training: 4 × 6–8 reps, 80% 1-RM, 2.5-min rest between sets. Tempo: 2 s concentric; 4 s eccentric Control: No training	Training group: single session of heavy load wrist flexor strength training	n = 18, F = 8, M = 10	Females: 23.38 ± 3.29 years Males: 26.80 ± 9.60 years	SCE and SICI via TMS	Heavy load strength training: CSE ↑ 48.05 ± 60.17%, SICI ↓ 26.85 ± 3.85% Control: CSE ↑ 8.4 ± 10.38%, SICI ↓ 2.16 ± 6.11%	8
Siddique et al., 2020 [24]	RCT	II	MPST: 4 × 6–8 reps at 80% 1-RM; Tempo: 1 Hz, 3 s concentric, 4 s eccentric SPST: 4 × 6–8 reps at 80% 1-RM; Tempo: self paced Isometric strength training: 4 × 6–8 reps at 80% MVIC, 7 s hold per rep Control group: No training	Training groups: 3×/week for 4 weeks of strength training of the biceps brachii	n = 42, F = 20, M = 22	25.1 ± 5.8 years	CSE and SICI via TMS, strength via 1-RM	MPST: 1-RM strength ↑ 18%, CSE ↑ 113%, SICI ↓ 60% SPST: 1-RM strength ↑ 25%, CSE and SICI ↔ Isometric: 1-RM strength ↑ 3%, CSE and SICI ↔ Control: 1-RMstrength ↑ 7%, CSE and SICI ↔	7
Weier et al., 2012 [25]	RCT	II	Strength training: 4 weeks of heavy load squat strength training, 4 sets × 6–8 reps at 80% of 1-RM. Tempo: 3 s concentric—3 s eccentric Control: No training	All training groups: 4 weeks of heavy load squat strength training, 3×/week for 4 weeks	n = 12; F = 6, M = 6	Aged 18–27 years; ST: 20 ± 0.8 years, Control 22 ± 0.6 years	CSE and SICI via TMS	Strength training: 87% ↑ in 1 RM squat strength; CSE ↑ 116%, SICI ↓ 32% Control: CSE and SICI: ↔	7

Note: MPST refers to metronome paces strength training; SPST refers to self-paced strength training; CSE referes to corticospinal excitability; SICI refers to short-interval cortical inhibtion; TMS refers to transcranial magnetic stimulation.

## Appendix B

**Table A2:** PEDro Scale Modified from PEDro Physiotherapy Evidence Database.

Criteria	No	Yes	Where
**Eligibility criteria were specified**			
**Subjects were randomly allocated to groups**			
**Allocation was concealed**			
**The groups were similar at baseline regarding the most important prognostic indicators**			
**There was blinding of all subjects**			
**There was blinding of all therapists who administered the therapy**			
**There was blinding of all assessors who measured at least one key outcome**			
**Measures of at least one key outcome were obtained from more than 85% of the subjects initially allocated to groups**			
**All subjects for whom outcome measures were available received the treatment or control condition as allocated or, where this was not the case, data for at least one key outcome was analyzed by “intention to treat”**			
**The results of between-group statistical comparisons are reported for at least one key outcome**			
**The study provides both point measures and measures of variability for at least one key outcome**			
